# Factors associated with excessively lengthy treatment of tuberculosis in the eastern Paris region of France in 2004

**DOI:** 10.1186/1471-2458-10-495

**Published:** 2010-08-18

**Authors:** Nadia Valin, Gilles Hejblum, Isabelle Borget, Henri-Pierre Mallet, Fadi Antoun, Didier Che, Christos Chouaid

**Affiliations:** 1 AP-HP, Hôpital Saint-Antoine, Service de Maladies Infectieuses et Tropicales, Paris, F-75012 France; 2 AP-HP, Hôpital Saint Antoine, Unité de Santé Publique, Paris, F-75012 France; 3 INSERM, U707, Paris, F-75012 France; 4 Direction de l'Action Sociale de l'Enfance et de la Santé, Cellule Tuberculose, Paris, F-75020 France; 5 UPMC Univ Paris 06, UMR S 707, Paris, F-75012 France; 6 Institut de Veille Sanitaire, Saint-Maurice, F-94415 France; 7 AP-HP, Hôpital Saint-Antoine, Service de Pneumologie, Paris, F-75012 France

## Abstract

**Background:**

Few data are available on prescriber adherence to tuberculosis (TB) treatment guidelines. In particular, excessively long treatment carries a risk of avoidable adverse effects and represents a waste of healthcare resources. We examined factors potentially associated with excessively long treatment.

**Methods:**

We reviewed the medical records of patients diagnosed with TB in 2004 in the eastern Paris region. Sociodemographic and clinical factors associated with excessively long treatment were identified by logistic regression analyses. Based on contemporary guidelines, excessively long treatment was defined as more than 6 months of a four-drug regimen for thoracic TB with full sensitive strains, and more than 12 months for patients with extrathoracic TB.

**Results:**

Analyses concerned 478 patients with a median age of 36.0 ± 13.5 years, of whom 48% were living in precarious conditions (i.e. poor living conditions and/or no health insurance), 80% were born abroad, and 17% were HIV-seropositive. TB was restricted to the chest in 279 patients (isolated pulmonary, pleuropulmonary, and isolated pleural TB in 245, 13, and 21 patients, respectively), exclusively extrathoracic in 115 patients, and mixed in the remaining 84 patients. Treatment was prescribed by a chest specialist in 211 cases (44.1%) and 295 patients (61.7%) were managed in a single institution. The treatment duration complied with contemporary guidelines in 316 cases (66.1%) and was excessively long in 162 cases (33.9%). The median duration of excessively long treatment was 313 days (IQR: 272-412). In multivariate analysis, isolated thoracic TB, previous TB, HIV infection, a prescriber other than a chest specialist, and management in more than one healthcare center during treatment were independently associated with excessively lengthy treatment.

**Conclusion:**

One-third of TB patients received excessively long treatment, reflecting inadequate awareness of management guidelines or unwillingness to implement them.

## Background

Many practitioners fail to comply with clinical practice guidelines[1]. In the United States for example, one study showed that a quarter of physicians prescribed excessively lengthy tuberculosis (TB) therapy, even though many of them were aware of the relevant guidelines[2]. Likewise, a French study based on a self-questionnaire completed by practitioners compared actual practices with TB management guidelines[3]. These practitioners declared that they frequently prescribed lengthy treatments to patients with extrapulmonary TB. Such excessive treatment carries a risk of avoidable adverse effects and represents a waste of healthcare resources. Here we examined the frequency of and risk factors for excessively lengthy TB therapy among patients diagnosed in the eastern Paris region of France in 2004.

## Methods

### Patients

All patients diagnosed with *Mycobacterium tuberculosis *culture-positive TB (thoracic, pulmonary or pleural, and/or extrathoracic) between 1 January and 31 December 2004 in the eastern Paris region were considered for inclusion in this retrospective study. Sociodemographic and clinical data were collected from the medical files, as previously described[4].

We excluded patients whose treatment period was not known, patients with multidrug-resistant TB (for whom the recommended treatment period is at least 18 months), and patients whose treatment was too short (less than 6 months of a four-drug regimen or of a three-drug regimen including pyrazinamide, or less than 9 months of a three-drug regimen without pyrazinamide). These latter patients are described in detail elsewhere, along with factors associated with excessively short treatment[4].

The remaining patients were divided into those whose treatment period complied with contemporary guidelines and those whose treatment was excessively lengthy, taking into account the drug regimen and the form of TB.

### Treatment duration according to international and French guidelines

WHO guidelines contemporary to the study recommended a 6-month four-drug regimen for newly diagnosed extrapulmonary and pulmonary TB[5,6]. The corresponding French guidelines specified that treatment for minor forms of extrapulmonary TB should be as long as that for pulmonary TB, but a 9- to 12-month course could be prescribed for severe extrapulmonary or neuro-meningeal TB[7]. Based on the French guidelines, we considered that a 6-month four-drug regimen for pulmonary-TB and a 6- to 12-month course for extrapulmonary TB were in keeping with guidelines. In addition, 6 months of a three-drug regimen including rifampicin, isoniazid and pyrazinamide for patients with fully sensitive strains, and 9 months of a three-drug regimen without pyrazinamide were also considered to be in keeping with guidelines. In case of resistance to isoniazid, isoniazid was replaced by ethambutol and treatment could be extended to 12 months[5-7]. All other patients with no prior history of TB were considered to have received excessively lengthy therapy.

In 2003, WHO recommended an 8-month retreatment regimen with first-line drugs while awaiting drug susceptibility testing (DST) results for patients who had relapsed[5]. In France, DST being readily available and rates of MDR-TB being low, contemporary guidelines recommended prompt empirical therapy with first-line drugs while waiting for the results of DST: longer treatment for previously treated patients was not routinely recommended[7]. Based on French guidelines, we considered that a 6-month course of standard treatment was in keeping with guidelines in patients with fully sensitive strains who had previously been treated for TB.

As treatments rarely last precisely the recommended period in practice, periods of 167-213 days were considered to correspond to six months, 213-274 days to 6-9 months, 274-365 days to 12 months, and 366-543 days to 18 months.

The study protocol was approved by the Saint Antoine Teaching Hospital Ethics Committee.

### Statistical analysis

We analyzed sociodemographic and clinical characteristics (age, sex, place of birth, socioeconomic status, previous TB, main comorbidities, form of TB, and drug resistance) and TB management modalities (specialty of the prescribing physician, the healthcare center(s) where follow-up took place, the type and number of healthcare structures involved in the management of individual patients, use of directly observed therapy (DOT), and computerized patient follow-up). A precarious socio-economical situation was defined as the absence of health insurance and/or poor living conditions (e.g. homelessness, living in a migrant shelter, or living in a hostel). The number of healthcare institutions managing a given patient was defined as the number of different physicians or healthcare structures involved in the patient's follow-up (e.g. general practitioner, hospital department or convalescence unit).

Stata software version 8 (StataCorp LP, TX) was used for all analyses, and significance was assumed at p < 0.05. Fisher's exact test was used to identify significant differences between categorical variables, and the Wilcoxon-Mann-Whitney test was used for continuous variables. Logistic regression analysis was used to identify predictors of excessively lengthy therapy. All variables with p values ≤ 0.20 in univariate analysis were included in multivariate analysis, with backward elimination of the variables.

## Results

**Figure 1:**
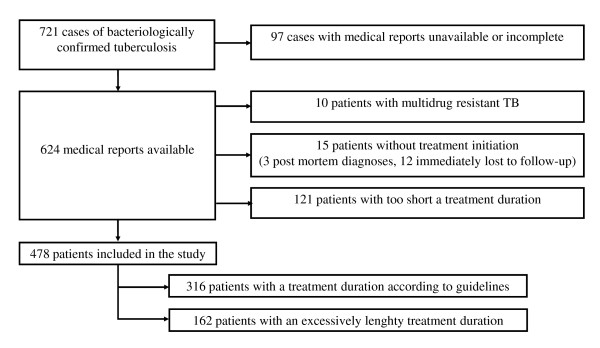
**Study profile**.

A total of 721 cases of bacteriologically confirmed TB were diagnosed in the eastern Paris region in 2004. The medical files of 97 patients (13.4%) did not include the treatment duration follow-up data, or could not be found despite an active search, and 15 patients (2.1%) had a post-mortem diagnosis or were immediately lost to follow-up and therefore received no treatment (Figure 1). Ten patients had multidrug-resistant TB, and 121 patients were treated for less than 6 months. The reasons for excessively short treatment were loss to follow-up (65 patients, 53.7%), treatment interruption (18 patients, 14.9%), transfer to another healthcare structure (14 patients, 11.6%) and death (24 patients, 19.8%).

This analysis focused solely on the remaining 478 patients, 316 (66.1%) of whom had a treatment duration in keeping with contemporary guidelines, and 162 (33.9%) received excessively lengthy therapy.

These 478 patients were managed in 19 healthcare structures, comprising 10 university hospitals, three general hospitals, a detention center, and five welfare centers, in respectively 68.7%, 21.5%, 1.1% and 8.7% of cases.

Median age was 36.0 ± 13.5 years, 68% of patients were male and 80% were born abroad. The main sociodemographic, clinical and management characteristics of the 478 patients included in the study are summarized in Table 1 according to the treatment duration (according to guidelines/excessive). TB was restricted to the chest in 279 patients (isolated pulmonary TB in 245 patients, pleuropulmonary TB in 13 patients, and isolated pleural TB in 21 patients), exclusively extrathoracic in 115 patients, and mixed in the remaining 84 patients.

**Table 1:** Clinical and management characteristics of the study patients and analysis of features associated with an excessively lengthy treatment.

Feature	Treatment duration				Statistical analyses			
	**All patients** **N = 478**	**According to** **guidelines** **N = 316**	**Excessive** **duration** **N = 162**		**Univariate** **analysis**		**Multivariate** **analysis**	
	
			**Number of** **patients**	**Median duration** **[IQR]**	**Odds Ratio** **[95%CI]**	**P value**	**Odds Ratio** **[95%CI]**	**P value**
			
***Clinical characteristics***								
*Previous TB*	53 (11.1%)	22 (7.0%)	31 (19.1%)	315 [252-387]	3.0 [1.8-5.8]	<0.0001	2.9 [1.6-5.4]	0.001
*Isolated thoracic TB*	279 (58.4%)	172 (54.4%)	107 (66.0%)	275 [250-311]	1.6 [1.1-2.4]	0.02	2.2 [1.4-3.5]	<0.0001
*HIV seropositivity*	74 (16.9%)	36 (11.4%)	38 (23.5%)	361 [289-418]	2.3 [1.4-3.8]	0.001	2.5 [1.4-4.4]	0.002
*Alcoholism*	83 (19.4%)	42 (13.3%)	41 (25.3%)	287 [266-367]	2.0 [1.2-3.3]	0.004***	-	-
*Injecting drug use*	10 (2.1%)	02 (0.6%)	08 (4.9%)	352 [305-419]	7.5 [1.6-35.8]	0.01***	-	-
*Psychiatric disorders*	38 (7.9%)	23 (7.3%)	15 (9.3%)	321 [274-367]	1.2 [0.6-2.3]	0.62		
Precarious situation*	210 (43.9%)	137 (43.4%)	73 (45.1%)	292 [274-407]	1.0 [0.7-1.5]	0.98		
*Isoniazid resistance*	27 (5.6%)	17 (5.4%)	10 (6.2%)	273 [241-396]	1.1[0.5-2.6]	0.72		
***Management modalities***								
*Prescriber*					0.002			0.01
Chest specialist	211 (44.1)	157 (49.7%)	54 (33.3%)	274 [265-315]	1 (reference)		1 (reference)	
Infectious disease specialist	171 (35.8%)	98 (31.0%)	73 (45.1%)	354 [274-427]	2.2 [1.4-3.3]		2.1 [1.3-3.4]	
Other	96 (20.1%)	61 (19.3%)	35 (21.6%)	379 [287-498]	1.7 [1.0-2.8]		1.9 [1.1-3.3]	
*Number of institutions per patient*						0.01		0.001
1	295 (61.7%)	210 (66.7%)	85 (52.8%)	282 [257-375]	1 (reference)		1 (reference)	
2	152 (31.8%)	93 (29.5%)	59 (36.6%)	366 [274-448]	1.6 [1.0-2.4]		1.7 [1.1-2.7]	
≥3	29 (6.5%)	12 (3.8%)	17 (10.6%)	356 [292-471]	3.5 [1.6-7.6]		4.3 [1.9-10.1]	
Place of follow-up**						0.07***		-
University hospital	423 (88.5%)	274 (86.7%)	149 (92.0%)	332 [271-431]	1 (reference)			
Welfare centers or other	55 (11.5%)	42 (13.3%)	13 (8.0%)	276 [273-288]	1.7 [0.9-3.4]			
*Directly Observed Therapy*	32 (6.7%)	16 (5.1%)	16 (9.9%)	292 [275-352]	2.0 [1.0-4.2]	0.06***	-	-
*Computerized follow-up*	145 (30.3%)	104 (34.2%)	41 (25.3%)	294 [274-367]	0.7 [0.4-1.0]	0.08***	-	-

*Poor living conditions and/or no health insurance

**Main structure used by each patient

***Non significant variables in the multivariate analysis

The treatment durations for isolated thoracic TB and extrathoracic TB are detailed in Table 2. Among the 279 patients with isolated thoracic TB, 107 (38.3%) had excessively lengthy therapy; 23 of these patients were HIV-seropositive, including a patient who received a three-drug regimen without pyrazinamide. Fifty-five patients with extrathoracic TB (27.6%) were treated for excessively lengthy periods (more than 12 months). Respectively 65.3%, 90.9% and 85.4% of patients with nodal, osteoarticular and disseminated forms were treated for excessively lengthy periods (more than 6 months). In univariate analyses (right part of Table 1), the following factors were associated with excessively lengthy therapy: isolated thoracic TB (p = 0.02), HIV seropositivity (p = 0.001), injecting drug use (p = 0.01), alcoholism (p = 0.004), previous TB (p < 0.0001), treatment by a physician other than a chest specialist (p = 0.002), and use of more than one healthcare structure during follow-up (p = 0.01). In multivariate analysis (Table 1), isolated thoracic TB, HIV seropositivity, previous TB, a prescriber other than a chest specialist, and use of more than one healthcare structure during follow-up remained significantly associated with excessively lengthy treatment.

**Table 2:** Treatment duration for isolated thoracic tuberculosis (TB) according to the type of treatment, and for extrathoracic TB according to the disease location.

Type of patients	Treatment duration (months)				
	**6**	**6-9**	**9-12**	**12-18**	**> 18**
	
*Isolated thoracic TB, N = 279*	163 (58.4%)*	*46 (16.5%)*	*58 (20.8%)*	*10 (3.6%)*	*2 (0.7%)*
Four-drug regimen, N = 241	146 (60.6%)	**30 (12.5%)**	**53 (22.0%)**	**10 (4.1%)**	**2 (0.8%)**
Three-drug regimen without ethambutol, N = 23	14 (60.9%)	**7 (30.4%)**	**2 (8.7%)**	-	-
Three-drug regimen without pyrazinamide, N = 8	3 (37.5%)	2 (25.0%)	**3 (37.5%)**	-	-
Four drug regimen and isoniazid resistance, N = 7		7 (100%)			
*Extrathoracic TB N = 199*	*53 (26.6%)*	*34 (17.1%)*	*57 (28.7%)*	*44 (22.1%)*	*11 (5.5%)*
Meningeal, N = 7	2 (28.6%)	0	2 (28.6%)	**3 (42.8%)**	-
Isolated nodal, N = 98	34 (34.7%)	18 (18.4%)	29 (29.5%)	**13 (13.3%)**	**4 (4.1%)**
Isolated osteoarticular, N = 22	2 (9.1%)	2 (9.1%)	6 (27.3%)	**9 (40.9%)**	**3 (13.6%)**
Disseminated or military, N = 41	6 (14.6%)	4 (9.8%)	11 (26.8%)	**16 (39.0%)**	**4 (9.8%)**
Other, N = 31	9 (29.0%)	10 (32.3%)	9 (29.0%)	**3 (9.7%)**	-

Abnormally lengthy treatments are in bold.

## Discussion

In 2004, in the eastern Paris region of France, almost one-third of patients diagnosed with TB were treated for periods exceeding those recommended by contemporary French and WHO guidelines[5,7]. These results are in keeping with some other recent reports. For example, a 2004 questionnaire-based survey of 66 prescribers of antituberculous treatment in France showed that pulmonary TB was treated for an average of 6 months, miliary and nodal forms for 9 months, and osseous forms for 12 months,[3] while the respondents recommended nine months of treatment (range 6-18 months) for HIV-infected patients with pulmonary TB. This tendency to treat for longer than recommended has also been reported in the United States, where one study showed that 28% of physicians exceeded the recommended treatment period[2]. Likewise, in India, 60.2% of prescriptions were for periods longer than necessary[8].

The main consequences of excessively lengthy treatment are higher costs and more frequent dose-dependent adverse effects, including peripheral neuropathies and liver toxicity, especially in patients with underlying liver disease[9,10]. Moreover, prolonged treatment can have a negative impact on patient adherence,[10] thereby increasing the risk of selecting resistant strains.

The reasons for these deviations from official recommendations are unclear. In France, possible reasons include the general tendency to overuse antiinfectives, the fear of not eradicating a potentially life-threatening infection, the fear of relapse, the incorrect belief that extrapulmonary TB is more difficult to treat than pulmonary TB, and simple unawareness of practice guidelines[3]. In our study, cases of MDR-TB cases were excluded, but drug susceptibility testing results were not available for nine patients who might therefore have been infected with MDR-TB, and the weight of these patients in the analysis results may be of concern. However, multivariate analysis excluding these nine patients yielded similar results, with the same variables remaining significant. When a control sputum smear is positive at the end of the intensive treatment phase, treatment prolongation might be warranted. However, French guidelines do not recommend such smear controls, except in the case of MDR-TB or if symptoms persist or reappear[7]. In our study, analysis of the medical reports confirmed that the practitioners had not prescribed such control smears. We found that infectious disease specialists were more likely than chest specialists to prescribe excessively long treatment, and that HIV-seropositive patients were also more likely to be prescribed excessively lengthy treatment (Table 1). WHO 2003 guidelines and French guidelines and previous studies recommended that TB patients living with HIV should receive the same TB treatment duration as HIV-seronegative patients[5-7,11]. However, infectious disease specialists often prefer to treat longer than recommended because they consider their patients more vulnerable, owing to their immunodepression and the relatively high frequency of disseminated TB and severe inflammatory forms[12]. A recent retrospective study of HIV-seropositive TB patients showed that those receiving a six-month course of treatment had a higher risk of relapse than patients treated for longer periods[13]. Current (i.e. 2010) WHO guidelines specify that some experts recommend prolonging TB treatment for persons living with HIV[14]. Therefore, the excessively long treatment of HIV-infected patients in our study probably reflects the evolution of practices and guidelines.

A prior history of TB was also associated with excessively lengthy treatment (Table 1), even though a six-month course is considered adequate for patients with fully sensitive strains[3]. Excessively lengthy treatment was more frequent among patients with isolated thoracic TB than among those with extrathoracic TB, but we found that a substantial number of patients with extrathoracic TB due to sensitive strains were treated for more than 12 months, conflicting with international guidelines and published data[5-7]. We did not consider a 12-month course excessive for extra-pulmonary TB, as international guidelines specify that "treatment can be prolonged to 9 or 12 months in case of severe extrapulmonary or neuromeningeal TB"[7]. However, contemporary French guidelines did not recommend special treatment for patients with extrathoracic forms[7]. Assuming that 6 months of treatment is adequate for all forms of extra-pulmonary TB, with the exception of neuromeningeal TB, more than half of the patients with nodal, osteaoarticular and disseminated forms received excessively long treatment in our study. Similar results have been obtained elsewhere,[2] calling for better training of prescribers or, alternatively, for treatment of these forms solely in specialized centers.

Patients who were managed in several healthcare structures were more likely to receive excessively long therapy. As the date of treatment initiation may not always be available for the second or third practitioner dealing with a given patient, these practitioners may prefer to err on the side of excessively long therapy. Centralized computer records for use by all national healthcare structures might improve the quality and conformity of patient care. Indeed, in a pediatric study, the use of computers improved the recording and compliance of management plans, although doctors found the system "too tedious to use during routine care"[15]. In France, such software has been shown to improve TB patient care and to reduce losses to follow-up[16,17].

To our knowledge, ours is the first study to analyze determinants of excessively lengthy TB treatment in a low-incident country. Most previous studies of TB patient management focused on patient-related factors involved in excessively short treatment, such as alcohol dependence, drug use, and precarious living conditions[18-20]. Our findings show that excessively lengthy treatments are mainly associated with patient management modalities. This is also the first study to examine actual treatment periods based on patients' medical reports rather than on an audit of physician practices[3].

This study is limited by its retrospective nature, including the fact that some patients were excluded because their files could not be found despite an extensive archive search. The study was conducted solely in the eastern Paris region, and included five of the French teaching hospitals that notify the largest number of TB cases (between 50 and 100 per year). Management practices might be somewhat different in smaller healthcare structures. However, it has been reported that physicians who treat few TB patients are less likely to be aware of TB management guidelines and that they tend to treat TB for far longer periods[21].

## Conclusion

This study shows that one-third of TB patients received excessively lengthy treatment in 2004 in the eastern Paris region of France. These results call for programs to improve physician awareness of TB management guidelines and to improve adherence to these guidelines, especially with respect to HIV-seropositive patients. A national tuberculosis control program launched in France in July 2007 aimed at proposing control strategies. In particular, monitoring of the treatment duration is expected to result in more appropriate treatment periods, in keeping with current guidelines[22]. Further evaluation of such data should allow us to detect the changes that have occurred since 2004.

## Competing interests

The authors declare that they have no competing interests.

This research was supported by Fondation de France and Comité National contre les Maladies Respiratoires.

## Authors' contributions

NV, CC and GH had the original idea for the paper, performed the literature search and contributed by assisting with conception and design, data acquisition, analysis of data, and drafting of the manuscript. NV performed statistical analysis. IB contributed to this work by assisting in data acquisition. The remaining authors made substantial contributions to the conception and design, data acquisition or interpretation of data, and critically revised the intellectual content of the manuscript. The final version of the paper was approved by all the authors.

## Pre-publication history

The pre-publication history for this paper can be accessed here:

http://www.biomedcentral.com/1471-2458/10/495/prepub
